# *In vitro* spleen cell cytokine responses of adult mice immunized with a recombinant PorA (major outer membrane protein [MOMP]) from *Campylobacter jejuni*

**DOI:** 10.1038/s41598-019-48249-3

**Published:** 2019-08-19

**Authors:** M. John Albert, Raj Raghupathy, Islam Khan, Fawaz Y. Azizieh

**Affiliations:** 1 Department of Microbiology, Faculty of Medicine, Kuwait University, Jabriya, Kuwait; 2 Department of Biochemistry, Faculty of Medicine, Kuwait University, Jabriya, Kuwait; 3 Department of Mathematics and Natural Sciences, International Centre for Applied Mathematics and Computational Bioengineering, Gulf University for Science and Technology, Mishref, Kuwait

**Keywords:** Immunology, Interleukins

## Abstract

There is no information on cytokine profiles for use as markers of protection in *Campylobacter jejuni* infection. To study this, we used outer membrane protein (MOMP [PorA]) as the vaccine for protection and spleen cell cytokines as markers of protection. We cloned and expressed *porA* from *C*. *jejuni*111 and immunized mice by the intraperitoneal route. Subsequently, mice were orally challenged with live *C*. *jejuni* 111. The vaccine induced protection as evidenced by reduced fecal excretion of *C*. *jejuni*111. Cytokines were measured *in vitro* after stimulation of spleen cells with MOMP. The levels of pro-inflammatory cytokines, IL-12, TNF-α, IL-17A and IL-17F were similar in control and test mice. The levels of pro-inflammatory cytokines, IL-2 and IFN-γ were higher in control mice than in test mice, and the levels of pro-inflammatory cytokines, IL-8 and IL-1β were higher in test mice than in control mice. Among the two anti-inflammatory cytokines, the levels were similar for IL-10 but higher for IL-4 in test mice than in control mice. Ratios of pro-inflammatory to anti-inflammatory cytokines showed a bias towards an anti-inflammatory response in favor of antibody production reflecting the role of antibodies in immunity. Cytokine production patterns by spleen cells may be used as markers of protection in the mouse model.

## Introduction

*Campylobacter jejuni* is a major foodborne pathogen and a cause of diarrhea worldwide including in Kuwait^1,2^. Control and prevention of *C*. *jejuni* diarrhea through vaccination is a priority^3^. Whole cell and subunit antigens of *C*. *jejuni* have been tested as potential candidate vaccines. These are live attenuated vaccines, killed whole cell vaccines, and subunit vaccines that include flagellar components, outer membrane protein and capsular polysaccharides^4–6^. One such subunit vaccine is a fusion protein – major outer membrane protein (MOMP or PorA) of *C*. *jejuni* fused with a carrier protein, glutathione S transferase (GST). The fusion protein is GST-PorA^7^. On oral immunization, GST-PorA imparted protection in an adult mouse intestinal colonization model of infection^8^. The side effects of a carrier protein such as GST in humans are not known if GST-PorA were to be used as a potential human vaccine. Therefore, we evaluated a recombinant PorA (MOMP) alone as a potential vaccine candidate in the adult mouse colonization model. There are numerous studies which investigated the roles of whole live or dead *C*. *jejuni* or various components such as lipooligosaccharide (LOS), flagellum and cytolethal distending toxin (CDT) of *C*. *jejuni* in inducing cytokines in both *in vivo* and *in vitro* models of infection^9^. But there are no studies which investigated the role of MOMP of *C*. *jejuni* on cytokine production. MOMP is present in abundant quantity on bacteria and is a surface structure which interacts with various environments with which the bacteria come into contact. Therefore, we also measured selected pro-inflammatory and anti-inflammatory cytokines *in vitro* in the spleen cells from mice immunized with MOMP to investigate how immunization influences their levels and whether their levels can be used as predictors of immunity. There is a link between cytokines and immunity as development of immunity is mediated by production of cytokines^10^.

## Materials and Methods

All methods were carried out in accordance with relevant guidelines and regulations.

### Bacteria and culture conditions

*C*. *jejuni* strain 111 (Penner serotype O:1,44) was cultured from the stool of a diarrheal patient in Kuwait. It was found to colonize mouse intestine in previous studies^8,10^. Stock culture was maintained in Brucella broth (Becton & Dickinson, Sparks, MD, USA) with 15% (vol/vol) glycerol at −70 °C. The stock culture was revived on agar with 5% defibrinated sheep blood (Oxoid, Basingstoke, Hampshire, England) and incubated at 42 °C for 48 h in a microaerobic atmosphere generated by Campigen (Oxoid). The identity of the bacteria was confirmed by cultural characteristics and molecular methods^11^.

### Preparation of enriched MOMP (eMOMP)

The MOMP of *C*. *jejuni* 111 was enriched by the Sarkosyl method^12^. Briefly, the bacteria were grown on blood agar at 42 °C for 48 h in a microaerobic atmosphere. Bacterial cells were disrupted by sonication and centrifuged at 5000 × g to remove whole cells. The supernatant was centrifuged at 100, 000 × g for 1 h at 4 °C in an L8-70 ultracentrifuge (Beckman, Fullerton, CA, USA). The resultant pellet was then treated with sodium lauryl sarcosinate (Sigma, St. Louis, MO, USA). The Sarkosyl-insoluble portion was used as the eMOMP.

### Ethics approval

Animal studies were approved by the Animal Ethics Committee of the Health Sciences Center, Kuwait University, Kuwait (approval number, VDR/HSC/3429). Methods were carried out in accordance with the relevant guidelines and regulations.

### Production of rabbit antibodies to eMOMP of *C. jejuni* 111

The eMOMP preparation was separated by discontinuous sodium dodecyl sulfate-polyacrylamide gel electrophoresis (SDS-PAGE) with a 5% stacking gel and a 12.0% separating gel according to the method of Laemmli^13^, and stained with Coomassie blue. The protein band corresponding to the MOMP (~ 45-kDa) was excised from the gel. The band was homogenized in PBS (pH 7.2) and approximately 1 ml of the gel suspension containing 50 µg of the protein was mixed with an equal volume of incomplete Freund’s adjuvant (Sigma) and injected subcutaneously into an adult New Zealand White rabbit. This first dose was followed by two additional doses at 2-week intervals. The rabbit was bled three weeks after the last injection^14^.

### Cloning and expression of *porA* gene

Chromosomal DNA was purified from blood agar grown *C*. *jejuni* 111 using UCP pathogen Mini Kit (Qiagen, Germantown, MD, USA). *porA* gene was amplified with forward primer E1: 5′ ATG GAT CCA CTC CAC TTG AAG AA G CG 3′ and reverse primer R3: 5′ATG GTA CCG AAT TTG TAA AGA GCT TGA AG 3′ with attached restriction sites for *Bam*HI and *Kpn*I respectively (underlined)^14^ and a high-fidelity platinum Taq polymerase (Invitrogen, Carlsbad, CA, USA). These primers are expected to amplify the *porA* gene without the region encoding the signal peptide. The cycling conditions were 95 °C/3 min, followed by 25 cycles of 95 °C/30 s, 60 °C/30 s, 72 °C/1 s with a last extension at 72 °C/8 min. The PCR product was separated by electrophoresis on 1% agarose gel and visualized by ethidium bromide staining. The PCR product was digested with *Bam*HI and *Kpn*I enzymes and purified with QIAquick PCR Purification Kit (Qiagen). The cloning vector pQE-30 which expresses the target protein with a His_6_ tag at the N-terminus (Qiagen) was linearized by double-digestion with the same two enzymes, separated on 0.9% agarose gel and purified by using QIAquick gel extraction kit (Qiagen). The *porA* gene was ligated to the digested vector with T4 DNA ligase (Promega, Madison, WI, USA) and used to transform competent *Escherichia coli* M15 (pREP4) cells (Qiagen). Transformants were selected on Luria agar containing ampicillin (100 µg/ml) and kanamycin (25 µg/ml). Random colonies were grown in Luria broth with ampicillin and plasmid extracted with a plasmid extraction mini kit (Qiagen). The plasmid DNA was digested with *Bam*HI and *Kpn*I enzymes and separated by electrophoresis on 1% agarose gel.

A 2 h culture of *porA* recombinant *E*. *coli* clone in Luria broth with ampicillin (100 μg/ml) and kanamycin (25 μg/ml) was induced with 1 mM IPTG (isopropyl-β-D-thiogalactoside) (Sigma) for 2 h at 37 °C in a shaker incubator. The proteins from lysed bacterial pellet were analyzed by Western blot. For Western blotting, the separated proteins by Laemmli method as above, were transferred electrophoretically on to a nitrocellulose membrane (Bio-Rad, Hercules, CA, USA) and then blocked with 5% bovine serum albumin (BSA) (Sigma) in phosphate-buffered saline (PBS, pH 7.2) containing 0.1% Tween 20 (PBSTB). The membrane was reacted with an appropriate dilution of relevant rabbit polyclonal antibody (either His-tag antibody [Qiagen] or MOMP antibody) in PBST with 1% BSA (PBSTb). The secondary antibody, peroxidase-conjugated, AffiniPure, goat IgG anti-rabbit immunoglobulin, Fc fragment-specific (Jackson ImmunoResearch, West Grove, PA, USA), diluted 1 in 50,000, in PBSTb was added, after which the membrane was developed with enhanced-chemiluminescence Western blotting detection reagents according to the manufacturer’s instructions (Amersham Pharmacia Biotech, Little Chalfont, Buckinghamshire, United Kingdom).

Large-scale purification of rPorA (MOMP) preparation was carried out using Ni-NTA affinity column and elution with denaturing buffers containing 8 M urea by following the procedure supplied with the pQE-30 vector. Urea was eliminated by filtering through an Amicon ultracel 30-K ultra-4 centrifugal filter (Merck Millipore, Burlington, MA, USA). The concentration of the protein was measured by a Nanodrop 8000 instrument (Thermo Fisher Scientific, Waltham, MA, USA) and adjusted to 1 mg/ml with phosphate-buffered saline (PBS, pH 7.2). The preparation was sterilized by filtering through a disposable filter device with a 0.45 µm pore diameter polyethersulfone membrane (GE healthcare, Marlborough, MA, USA) and stored at −20 °C until used.

### Immunization and challenge of BALB/c mice

Six to eight weeks-old female BALB/c mice free of *Campylobacter* infection (tested by culturing stool by standard methods as above) were chosen from the Animal Resource Center of the Health Sciences Center, Kuwait University. Two days before the experiment, animals were separated and placed in individual cages. They were given a standard chow^8^ and water ad libitum throughout the experiment. Our preliminary studies showed that three doses (each dose containing 300 µg rPorA) given orally after neutralization of the gastric acidity by sodium bicarbonate, with an interval of a week between doses, and serum and intestinal antibody responses, tested a week after the third dose, did not result in an immune response to the antigen. We surmised that rPorA being a protein, was degraded by the enzymes of the digestive tract. Therefore, we decided to administer the antigen by the intraperitoneal (IP) route. Our preliminary studies showed that a dose of 100 µg of rPorA was an optimal dose without inducing toxic reaction in the animals. For protection studies using colonization model or for cytokine studies, mice were immunized by the IP route with PorA (100 µg per dose) three times at a weekly interval. Control mice were immunized with phosphate-buffered saline (PBS, pH 7.2).

A week after the third dose, mice for the colonization model study were orally challenged with 0.5 ml of live *C*. *jejuni* 111 in PBS containing 1 × 10^9^ CFU per ml. The preparation of bacteria and oral challenge were as described previously^15^. Fecal shedding of the challenge bacteria was monitored daily for 8 days as described previously^15^.

### Collection of feces and blood for antibody estimation

Five to seven fresh fecal pellets (equivalent to 100 to 200 mg) were collected from each mouse shortly before vaccination and a week after the third vaccine dose. To each mg of the feces, 100 µl of IgA extraction buffer (PBS containing 0.05% Tween 20, 0.5% fetal calf serum, 1 mg/ml ethylenediaminetetraactetic acid [EDTA], 1 mg/ml phenylmethylsulfonyl fluoride, and 200 µg/ml trypsin soybean inhibitor) (Sigma) was added. After 15 min of incubation on ice, fecal pellets were homogenized and centrifuged at 23,000 × g for 15 min at 4 °C. The supernatant was stored at −70 °C until assayed^15^. At the same times as the collection of feces, tail vein blood was collected. A 50 µl of blood were mixed with 950 µl of PBST. After one cycle of freezing and thawing, the sample was centrifuged at 400 × g for 15 min, and the supernatant was stored at −70 °C until assayed^15^.

### Measurement of total IgA in fecal extract

The total IgA in fecal pellets was determined by using an ELISA^16^. The wells of a Maxisorp immunoplate (Nunc, Rochester, NY, USA) were coated with affinity purified, human serum absorbed, goat anti-mouse IgA (Kirkegaard & Perry Laboratories, Gaithersberg, MD, USA) in carbonate buffer and incubated at 37 °C for 1 h followed by 24 h at 4 °C. The wells were washed three times with PBST and blocked with PBSTB at 37 °C for 2 h. Various concentrations (3 to 300 ng/ml) of purified mouse IgA (Bethyl Laboratories, Montgomery, TX, USA) or serial doubling dilutions (starting from 1 in 100) of fecal extracts (from pre-immunized and post-immunized mice) in PBSTb were added to the wells and incubated at 37 °C for 2 h. The wells were then washed three times with PBST and then incubated with horseradish peroxidase-conjugated, goat anti-mouse IgA antibody (Sigma) diluted to 1 in 5,000 in PBSTb at 37 °C for 2 h. Wells were washed three times with PBST, after which substrate, 2,2′-azino-di (3-ethyl-benzthiazoline sulfonate) (ABTS) (Sigma) was added. After 30 min at 37 °C, the optical density at 405 nm (OD_405 nm_) was measured in a Powerwave Microplate spectrophotometer (Biotek Instruments Inc., Vinooski, Vermont, USA). The amount of IgA present in a fecal sample was determined by interpolation of OD on a standard curve constructed with OD readings of known concentrations of purified mouse IgA. The pre-and post-immune samples were appropriately diluted so that they contained the same amount of IgA.

### Measurement of serum and fecal antibodies

Serum IgG and IgA antibodies and fecal IgA antibodies to PorA were assayed by enzyme-linked immunosorbent assays^15^. The wells of a Maxisorp immunoplate (Nunc) were coated with carbonate buffer (pH 9.6) containing 1 µg/ml of rPorA at 4 °C for 24 h. The wells were washed four times with PBST and blocked with PBSTB at 37 °C for 1 h. A 1: 100 dilution of lysed blood sample or a 1: 10 dilution of fecal IgA in PBSTb was added to the wells and allowed to react at 37 °C for 1 h. The wells were washed four times with PBST and reacted with horseradish peroxidase-conjugated goat anti-mouse secondary antibodies (that recognize IgG or IgA isotypes) (Kirkegaard & Perry Laboratories) diluted 1: 1000 in PBSTb at 37 °C for 1 h. The wells were washed four times with PBST. The substrate, ABTS (Sigma) was added, and after 30 min of incubation at 37 °C, OD_405  nm_ was measured.

In addition, agglutinating antibodies in feces were also assayed by the method of Ueki *et al*.^17^. Fifty ul of suspension of *C*. *jejuni* 111 in PBS (containing approximately 5 × 10^8^ CFU/ml) was added to each of a 96 V-shaped-well microtiter plate (Nunc), after which 50 µl of 2-fold serially diluted fecal IgA in PBS was added (with a starting dilution of 1: 10). After incubation at 37 °C for 2 h, the plate was kept at 4 °C for 20 h and the agglutination was read. The highest serum dilution showing agglutination was recorded as the titer.

### Stimulation of spleen cells from mice immunized intraperitoneally with PorA and control mice

A week after the third vaccine dose and immediately after collection of feces and blood as above, mice were killed by cervical dislocation. Abdominal area was sterilized with 70% alcohol. Spleen was removed through an incision. Connective tissues were removed and spleen rinsed in 70% alcohol and then in RPMI 1640 medium (Thermo Fisher Scientific) and placed in a petri dish with fresh RPMI 1640 medium. Spleen cells were teased out with forceps and forced through an 18 G needle and then through a 22 G needle three times each. The cells were transferred to a tube and centrifuged at 200 × g for 10 minutes to pellet the cells. Cells were washed twice by re-suspending in RPMI 1640 and centrifugation. The cell pellet was re-suspended in RPMI 1640 with 10% fetal bovine serum and 1% antibiotics (penicillin, streptomycin, gentamicin) (Thermo Fisher Scientific), cells were counted in a hemocytometer and diluted to 5 × 10^6^ cells per ml of the medium. Wells of a flat-bottomed microtiter plate (Nunc) were inoculated with 100 µl of cells and stimulated with 50 µl of PorA (final concentration of 10 µg/ml). Stimulation with the antigen was done in duplicate wells. The volume in each well was made up to 200 µl by adding 50 µl of RPMI 1640 medium. The plate was incubated in a 5% CO2 incubator at 37 °C for 144 h. For cytokine IL-4 only, stimulation with antigen was done for 24 h. At the end of the stimulation, supernatants were harvested and stored at −80 °C until assayed for cytokines.

### Cytokine assays

The following cytokines were assayed- pro-inflammatory cytokines: IFN-γ, TNF-α, IL-12, IL-8, IL-2, IL-17A and IL-17F and IL-1β; and anti-inflammatory cytokines: IL-10 and IL-4. All cytokines except IL-4 and IL-8 were assayed using a commercially available multiplex panel mouse cytokine assay kit (MTH17MAG-47K) (Merck Millipore, Darmstadt, Germany). This is a Luminex multiplex ELISA-based immunoassay containing fluorescent dyed beads coated with antibodies specific for cytokines (Luminex, Austin, TX, USA). Concentrations of the cytokines were measured using a MAGPIX array reader (Luminex) that quantifies cytokines using very small sample volumes. The cytokine concentrations were calculated using standard curves with a software provided by the manufacturer (Luminex Manager Software). The detection limits (pg/ml) of the cytokines were: IFN-γ, 1.1; TNFα, 2.3; IL-12, 3.9; IL-2, 1.0; IL-17A, 0.5; IL-17F, 0.5; IL-1b, 5.4; and IL-10, 2.0. Quality control tests were included as per the instructions of the manufacturer.

IL-8 after 144 h stimulation, and IL-4 after 24 h stimulation were assayed using respective sandwich ELISA kits according to manufacturer’s instructions (MyBiosource, San Diego, CA, USA). The cytokine concentration in the test sample was inferred by interpolating the optical density (OD) of the test well containing the sample to an OD curve constructed using standards with known quantities of the cytokine. The detection limits were 5 pg/ml for IL-8 and 1 pg/ml for IL-4.

### Statistics

Median levels of cytokines, antibody levels and amount of organisms excreted were compared by Mann-Whitney U test by SPSSS statistics (Chicago, IL, USA). Bacterial colonization rates were compared by Fisher’s exact test. A *P* value of ≤ 0.05 was considered significant.

## Results

### Cloning and expression of *porA* gene

**Figure 1:**
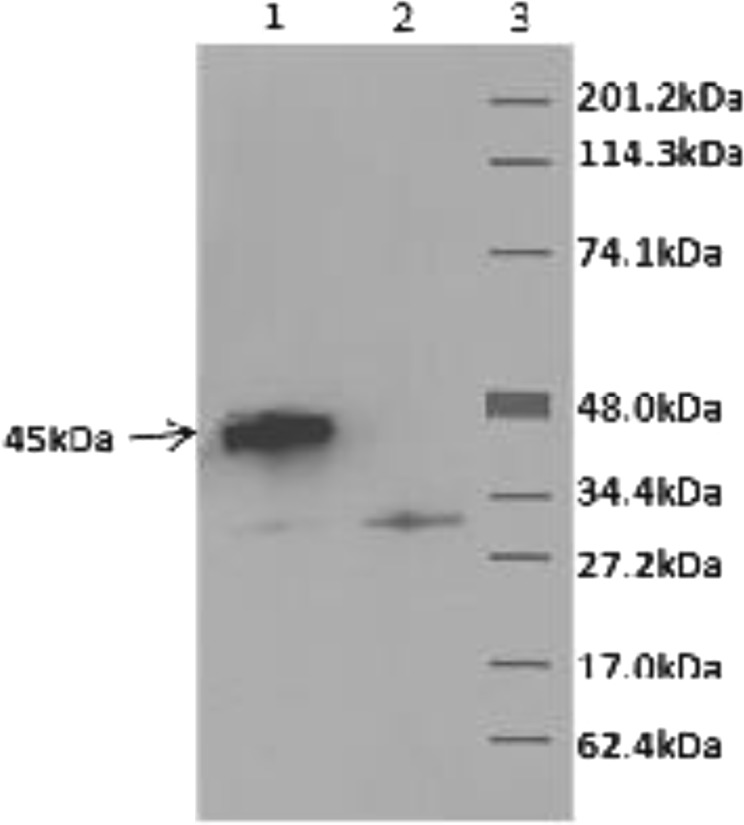
A representative Western blot of induced proteins. Induced proteins from *porA* clone (lane 1) and *E*. *coli* host (lane 2) were separated by SDS-PAGE, transferred to a nitrocellulose membrane and probed with *C*. *jejuni* 111 anti-MOMP antibody. The reaction was developed with enhanced-chemiluminescence detection system (Amersham Pharmacia Biotech). Note a prominent band of about 45-kDa in lane 1 (arrowed), which is absent in lane 2 (samples in lanes 1 and 2 were run on the adjacent wells of the same gel). Lane 3 has molecular weight markers labelled as per markers from Bio-Rad.

The *porA* gene was amplified from *C*. *jejuni* 111. It had 1209-bp and the sequence was identical to that of *C*. *jejuni* strain 01–0949 without the first 66-bp corresponding to the N-terminal 22 amino acids constituting the signal peptide (Accession number CP010301.1). The nucleotide identity of *porA* gene of strain 111 varied from 94 to 100% with those of the 101 *C*. *jejuni* strains deposited in the National Center for Biotechnology Information (https://blast.ncbi.nlm.nih.gov/Blast.cgi). No similarity with *porA* gene of other bacteria was found. The expressed protein from *porA* gene of strain 111 had a molecular weight of approximately 45-kDa (corresponded with MOMP) and reacted with anti-His_6_ tag antibody (Invitrogen) and rabbit polyclonal antibody to gel-purified MOMP from *C*. *jejuni* (Fig. 1).

### Colonization and excretion of *C*. *jejuni* 111

Ten mice were immunized by the IP route by MOMP and an equal number of mice with PBS as controls. One control mouse died. The colonization rates in test and control mice for 8 days are shown in Table 1. Even though the colonization rate in test mice showed variation, it was lower for all days compared to in control mice, and the difference achieved significance for day 7. Excretion of challenge bacteria showed variation in both control and test mice, but was lower in test mice for all days and this was statistically significant for 4/8 days and for mean excretion (Table 2).

**Table 1:** Colonization rates of *C*. *jejuni* 111 in control mice and in test mice vaccinated intraperitoneally with MOMP.

Day	No. of control mice colonized/total no.	No. of test mice colonized/total no.	P value for difference
1	7/9	7/10	1.00
2	8/9	8/10	1.00
3	9/9	7/10	0.21
4	9/9	9/10	1.00
5	9/9	6/10	0.08
6	8/9	8/10	1.00
7	9/9	3/10	0.003
8	9/9	9/10	1.00
Mean	9/9	7/10	0.21

P value by Fisher’s exact test.

**Table 2:** Fecal excretion of *C*. *jejuni* 111 in control mice and in test mice vaccinated intraperitoneally with MOMP.

Day	Excretion (cfu/mg) (SD) in control mice	Excretion (cfu/mg) (SD) in test mice	P value for difference in excretion
1	2.7 × 10^4^ (1.8 × 10^4^)	1.1 × 10^4^ (1.3 × 10^4^)	0.5
2	1.2 × 10^4^ (1.1 × 10^4^)	1.1 × 10^4^ (9.0 × 10^3^)	0.96
3	3.0 × 10^4^ (1.4 × 10^4^)	2.1 × 10^3^ ( 2. 9× 10^3^)	0.004
4	4.1 × 10^4^ (3.7 × 10^4^)	1.2 × 10^4^ (1.5 × 10^4^)	0.022
5	4.9 × 10^4^ (2.7 × 10^4^)	4.4 × 10^3^ (1.1 × 10^3^)	0.002
6	2.8 × 10^4^ (2.9 × 10^4^)	1.3 × 10^4^ (1.6 × 10^4^)	0.28
7	8.1 × 10^3^ (6.8 × 10^3^)	8.8 × 10^2^ (1.8 × 10^3^)	0.008
8	3.1 × 10^4^ (2.7 × 10^4^)	1.3 × 10^4^ (1.1 × 10^4^)	0.31
Mean	2.8 × 10^4^ (1.3 × 10^4^)	8.4 × 10^3^ (5.0 × 10^3^)	0.011

cfu: Colony forming unit. SD: Standard deviation.

P value by Mann-Whitney U test.

### Antibody responses to *C*. *jejuni* MOMP

There were significant increases in serum and fecal antibodies as measured by ELISA in post-immune sera compared to pre-immune sera in test animals (Table 3). The ELISA antibody levels in pre-and post-immune samples in control mice were similar and negligible (data not shown). In test animals, pre-immune fecal mean agglutinating antibody titer was 0, whereas post-immune fecal mean agglutinating antibody titer was 58 (range, 20–80; standard deviation, 23.94) (P = 0.0002 by Mann-Whitney U test). The agglutinating antibody titers in pre-immune and post-immune fecal samples in control animals were 0.

**Table 3:** Antibody responses in test mice vaccinated intraperitoneally with MOMP.

Antibody class in sample	Mean (SD) OD readings in		P value for differences in OD readings
	Pre-immune sera	Post-immune sera	
Serum IgG	0.077 (0.003)	2.725 (0.272)	0.00018
Serum IgA	0.105 (0.017)	0.170 (0.045)	0.00152
Fecal IgA	0.111 (0.023)	0.198 (0.041)	0.00044

SD: Standard Deviation. OD: Optical Density. P value by Mann-Whitney U test.

### Cytokine production by splenocytes from mice immunized intraperitoneally with MOMP

**Figure 2:**
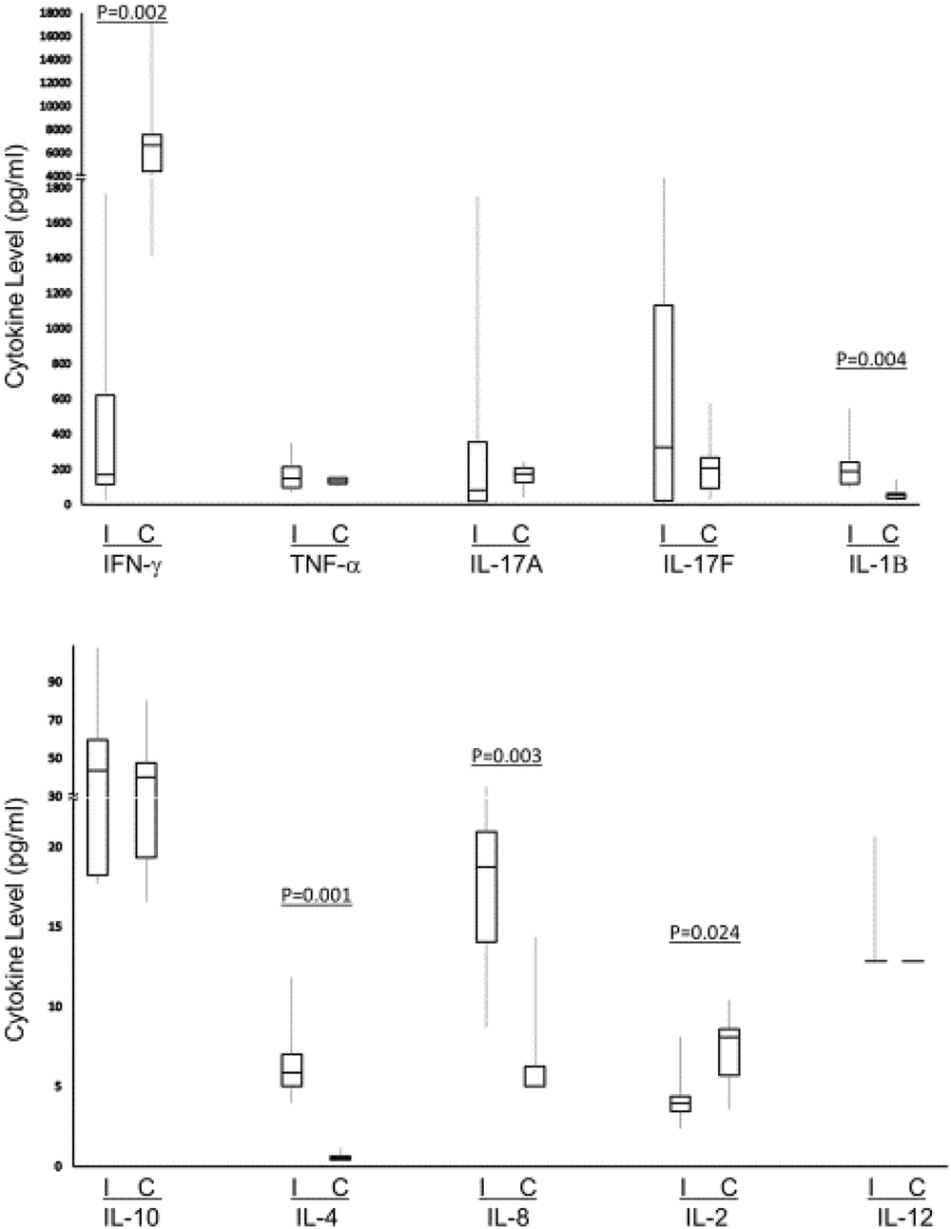
Levels of cytokines in the supernatants of splenocytes from control mice (C) and test mice immunized with MOMP (I). Lines in the boxplot indicate median values. P values (calculated by Mann-Whitney U test) for significantly different comparisons are indicated.

Eight mice were immunized with MOMP and seven mice with PBS. The test mice immunized with MOMP mounted good antibody responses in sera and feces similar to those seen in samples of test mice in colonization model of protection (data not shown). The levels of cytokines present in the supernatants of splenocytes from test and control mice after stimulation with MOMP are shown in Fig. 2. For pro-inflammatory cytokines, IL-12, TNF-α, IL-17A and IL-17F, the levels in control and test animals were similar. Among pro-inflammatory cytokines, the levels of IL-2 and IFN-γ were higher in the control mice than in test mice, and the levels of IL-8 and IL-1β were higher in test mice than in control mice. Among the two anti-inflammatory cytokines, the levels were similar for IL-10 but higher for IL-4 in test mice than in control mice. Differences in the ratios of levels of pro-inflammatory cytokines to levels of anti-inflammatory cytokines in immunized mice compared to in control mice are shown Table 4. The ratios of IL-12, IL-2, TNF-α, IL-17A and IL-17F to IL-10 were similar in test and control mice. The ratios of IFN-γ to IL-10, and those of IFN-γ, IL-12, IL-2, TNF-α, IL-17A, IL-17F and IL-8 to IL-4 were significantly higher in control mice than in test mice. The ratio of IL-8 to IL-10 was significantly higher in test mice than in control mice. Since many samples did not have measurable IL-1β, this cytokine was not included in the calculation of ratios.

**Table 4:** Differences in the ratios of pro-inflammatory cytokine levels to anti-inflammatory cytokine levels in immunized mice compared to in control mice.

Cytokine ration	Ratio in		P-value for difference
	Immmmunized mice	Control mice	
IFN-γ/IL-10	6.9	190.3	0.002
IL-12/IL-10	0.3	0.3	0.817
IL-2/IL-10	0.1	0.3	0.083
TNF-α/IL-10	4	3.8	1
IL-17A/IL-10	2.5	4.3	0.355
IL-17F/IL-10	8.5	5.2	0.908
IFN-γ/IL-4	33	12536	0.001
IL-12/IL-4	2.2	26.2	0.001
IL-2/IL-4	0.6	13.6	0.001
TNF-α/IL-4	26.4	243.3	0.001
IL-17A/IL-4	19.3	304.7	0.008
IL-17F/IL-4	76.2	290.6	0.064
IL-8/IL-10	0.4	0.3	0.008
IL-8/IL-4	3.1	12.5	0.001

P value by Mann-Whitney U test.

## Discussion

We have successfully cloned and expressed MOMP from *C*. *jejuni* 111. Since oral administration of MOMP did not elicit an antibody response because of possible degradation of the protein in the digestive tract, we immunized mice by the IP route. The IP route seems to have preserved the antigen as significant serum and fecal antibody responses were elicited. The intraperitoneally immunized animals were then subjected to oral challenge in the colonization model of infection. There was a reduced colonization rate of immunized mice. The immunized mice also excreted reduced amounts of *C*. *jejuni*. However, there was a variability in the colonization rate and excretion rate of the organism. This seems to be a feature of this mouse model for *C*. *jejuni* infection, as we have observed a similar trend when other immunogens were used^15,18^. However, our study suggested that immunization by the IP route imparted protection against *C*. *jejuni* infection. Therefore, we used this model to study cytokine responses by spleen cells, and whether spleen cell cytokine responses can be used as a marker of immunity.

The *porA* gene of strain 111 shares a very high homology with those from other strains. Homology among *porA* genes has been shown by another study^14^. Homology leads to sharing of common epitopes among MOMPs of different strains of *C*. *jejuni*. Shared epitopes impart heterologous protection^8^. Therefore, it is likely that MOMP expressed from the *porA* gene of strain 111 will offer cross-protection against heterologous strains.

Spleen is a lymphatic organ in the blood stream. The immune system of the spleen protects the body from invading pathogens. Innate and adaptive immune responses take place in the spleen^19^ and thus, generally the measurement of immune reactivity of splenocytes is a reflection of the immune responses in the host. Therefore, it was appropriate to study cytokine responses of the spleen cells after immunization of the animals and stimulation of the spleen cells by MOMP.

The levels of pro-inflammatory cytokines, IL-12, TNF-α, IL-17A and IL-17F in control and test animals were similar. However, the levels of pro-inflammatory cytokines, IL-2 and IFN-γ, were higher in control mice than in test mice, while the levels of the pro-inflammatory cytokines, IL-8 and IL-1β, were higher in test mice than in control mice. As for the two anti-inflammatory cytokines, the levels of IL-10 were similar in the two groups, but levels of IL-4 were higher in test mice than in control mice. Comparisons of pro-inflammatory to anti-inflammatory cytokine ratios between test mice and control mice reflected comparisons of absolute levels of cytokines in all instances except in two comparisons. IL-8 levels were higher in test mice and IL-12 levels were similar in both groups of mice, but their ratios with regard to IL-4 showed higher values for control mice.

In human volunteers orally vaccinated with killed *Campylobacter* whole cell (CWC) vaccine plus single mutant (m) LT (LTR192G), *in vitro* IFN-γ cytokine production by peripheral blood monocytes was deemed important for protection^20^. Similarly, in a human volunteer study of *C*. *jejuni* diarrhea, protection was associated with IFN-γ cytokine response by peripheral monocytes^21^. However, in our study, contrary to our expectation, splenocytes from immunized mice produced significantly less amount of IFN-γ compared to control mice. The differences with our studies are many including the type of vaccine, route of administration, the cells used for induction of cytokines and the species of experimental models. *C*. *jejuni* infection elicits Th1-promoting cytokines from APCs. Dendritic cells (DCs) increase the production of IL-2 after contact with and internalization of *C*. *jejuni*^22,23^. TNF-α is one of the cytokines that signals the early inflammatory response to *C*. *jejuni*. It is increased in the acute phase of gastroenteritis^24^ and in *in vivo* models of *C*. *jejuni* infection^25,26^. It is produced as early as 4–6 h by DCs^22^ and intestinal epithelial cells (IECs)^27^. IL-17A and IL-17 F are Th17 cytokines. Both Th1 cytokine (IFN-γ) and Th17 (IL-17) responses were seen in *in vivo* models during colonization and immune infiltration of intestine^26,28^. There is also increased infiltration of several immune cells. The number of apoptotic cells also increases^29^. IL-17A and IL-17F reduced the number of intracellular bacteria in an *ex vivo* model of infection using pediatric intestinal biopsies suggesting their importance in intestinal immunity^30^. Studies on the role of IL-2 in *C*. *jejuni* are rare. In a study where a human recombinant IL-2 was fed to mice, it resisted intestinal colonization with *C*. *jejuni*^31^. IL-8 also signals early inflammatory response to *C*. *jejuni*^24^. High levels of this cytokine were found in stool in acute stage of natural infection^32^. IECs produce this cytokine whose level continues to rise until 24–48 h post-infection^33^. Adherence/invasiveness of *C*. *jejuni* induces IL-8 from intestinal cells^34^. Killed bacteria also induce IL-8 from the monocytic cell line, THP-1^35^. CDT of *C*. *jejuni* induces IL-8 from IECs^36^. IL-8 plays a role in immune cell infiltration^37^. IL-1β is one of the cytokines of APCs and a promoter of pro-inflammatory response. High levels of this cytokine are present in the stools of patients with acute gastroenteritis^24,32^ and decreased during convalescence and recovery^24,38^. Its levels are increased in tissues in acute stage^25^. It is produced *in vitro* within 4 to 24 h from cultured DCs^22^ and macrophages^35^ on stimulation with killed *C*. *jejuni*. LOS of *C*. *jejuni* is an inducer of IL-1β^22^. The increased level of IL-1β coincides with accumulation of neutrophils and macrophages^25^ which will lead to exaggerated inflammation and pathology^26^. IL-10 controls inflammatory response. Infection of IL-10−/− mice with *C*. *jejuni* is characterized by increased levels of pro-inflammatory cytokines (TNF-α, IL-1β, IL-6, IFN-γ and IL-22) and infiltration of inflammatory cells in the colon^39,40^. IL-10 may also increase recruitment of Treg cells^39^. Concurrent production of IL-10 and pro-inflammatory cytokines (TNF-α, IL-6, IFN-γ) has been reported in tissue culture systems^41,42^. This is more so with live bacteria while sonicated bacteria produced less pro-inflammatory cytokines^41^. DCs and immune cells produce pro-inflammatory cytokines in response to *C*. *jejuni* infection^22^. IL-4 is a Th2 cytokine and is important for development of humoral immunity. It is produced in *in vitro* and *in vivo* models of infection^41,43^. DCs stimulated with LOS of *C*. *jejuni* can promote B cell proliferation in a T cell independent manner^44^. B cell responses are thought to be beneficial to control *C*. *jejuni* infection. IL-4 is involved in the resolution of pro-inflammatory response. Its level rose later in the infection which coincided with a decrease in the level of pro-inflammatory cytokines in many organs including spleen in a mouse lung model of infection^25,45^.

An interesting observation in this study is the bias towards a lower pro-inflammatory cytokine production pattern in immunized mice, based on the comparison of cytokine ratios. The ratios of IFN-α/IL-10, IFN-α/IL-4, IL-12/IL-4, IL-2/IL-4, TNF-α/IL-4, IL-17A/IL-4 and IL-8/IL-4 were all significantly lower, which is suggestive of a stronger bias towards anti-inflammatory Th2 cytokines and by corollary a stronger bias towards antibody production rather than cell-mediated immunity. A lower incidence of *Campylobacter* infection in breast-fed children in Mexico could be correlated with the presence of specific secretory IgA antibodies in mother’s milk^46^. In industrialized countries, there is a reduced incidence of *C*. *jejuni* diarrhea and increased levels of specific antibodies in chronic raw milk drinkers compared to first time drinkers^47^. Expatriate residents in Thailand were observed to have a reduced prevalence of *Campylobacter*-associated diarrheal illness related to a longer duration of residency with increased antibody levels^48^. In human volunteer studies, protection could be correlated with higher levels of serum and intestinal antibodies to the bacteria^49^. These observations suggested that immunization resulted in a shift towards an anti-inflammatory cytokine dominance in favor of humoral immunity. Feces of test mice contained significant titers of agglutinating antibodies. These fecal antibodies might have agglutinated *C*. *jejuni*, prevented colonization of the intestine and reduced the fecal bacterial load.

There are no studies which investigated the effect of MOMP on cytokine response. Our study suggested that on intraperitoneal immunization of adult mice, there is no alteration in the levels some cytokines (IL-2, TNF-α, IL-17A and IL-17F, IL-10), the levels of some cytokines are elevated (IL-4, IL-8 and IL-1β) and the levels of some cytokines are decreased (IL-2 and IFN-γ) in the spleen. The varying levels of these cytokines produced by spleen cells in this mouse model of infection may be used as markers of immunity when MOMP is used as the vaccine. We measured these cytokines one week after the 3^rd^ dose of the vaccine at which time we could demonstrate a significant antibody response and protection. The antibody response correlates well with the bias towards an anti-inflammatory or Th2 cytokine profile.

## Data Availability

All data generated or analyzed during this study are included in this published article.
